# An American Text-Book of Genito-Urinary Diseases, Syphilis and Diseases of the Skin

**Published:** 1898-06

**Authors:** 


An American Text-Book of Genito-Urinary Diseases, Syphilis and Diseases of the Skin. Edited by L. Botton Bangs, M. D., Consulting Surgeon to St. Luke's Hospital and the City Hospital, New York, and to the Methodist Episcopal Hospital, Brooklyn; Visiting Genito-urinary Surgeon to St. Mark's Hospital, New York; Late Professor of Genito-urinary and Venereal Diseases, New York Post Graduate Medical School and Hospital; and W. A. Hardaway, A. M., M. D., Professor of Diseases of the Skin and Syphilis in the Missouri Medical College, St. Louis; Physician for Diseases of the Skin to the Martha Parson's Hospital for Children and to St. John's Hospital, St. Louis. Illustrated with 300 engravings and 20 full-page colored plates. Philadelphia: W. B. Saunders, 925 Walnut street. 1898.

This work, which is designated An American Text-book of Genito-urinary Diseases, Syphilis and Diseases of the Skin, is, as the editors inform us, a collaboration of well-known authorities who have not been restricted in regard to the particular views set forth in its pages, but have been offered every facility for the free expression of individual opinion. It certainly sets forth the clinical facts and the principles of treatment, based on the results of modern research, serving not merely as a text-book for the student and practitioner, but also as a work of reference for the expert. It is essentially clinical and practical in its scope. The first subject treated in the work is on Urine analysis and a consideration of the urine in surgical diseases of the urinary tract, by Paul Thorndike, M. D., in which he describes the characteristics and chemical constituents of urine and the commoner and more practical methods for investigating them in a clear and concise manner, in which the various points are treated at a length proportionate to their intrinsic importance.

Diseases of the penis, by B. Farquhar Curtis, M. D., the contents of which are arranged as follows: 1. Absence, reduplication, etc.; 2. Hypospadias; 3. Epispadias; 4. Malformations and contractions of the meatus; 5. Malformations of the foreskin, phimosis and adhesions; 6. Paraphimosis; 7. Injuries; 8. Gangrene; 9. Inflammatory conditions; 10. Tuberculosis; 11. Varicose veins; 12. Neuralgia; 13. Priapism; 14. Tumors.

Of the individual articles there are so many that are excellent that it is difficult to select any for special mention. Diseases of the male urethra, by G. Frank Lydston, M. D., is comparatively brief, considering the importance and frequency of some of the complaints affecting this tract; however, the article is replete with information and one most valuable feature of it is the care and accuracy with which the subject is handled and especially that pertaining to therapeutics.

Diseases of the testicle and its coverings, the cord and the seminal vesicle, by Eugene Fuller, M. D. The article is essentially clinical in character, and gives the impression of being written direct from nature as seen in the consulting room and the hospital ward.

Diseases of the prostate, by J. William White, M. D., and Alfred C. Wood M. D. There is enough of the personal element in this article to give it individuality. The subject, treating on the hypertrophy of the prostate, receives much more than the usual attention. The therapeutical instructions given are full of detail and eminently practical.

Diseases of the bladder, by Edward Martin, M. D., and A. E. Tayler, M. D. Vesical calculus, by Francis S. Watson, M. D. Diseases of the ureter, by Christian Fenger, M. D., and S. C. Stanton, M. D. The surgical diseases of the kidney, by P. R. Bolton, M. D. Functional disordeis, by James Pederson, M. D., are the remaining individual articles to be found in the work under genito-urinary diseases. It would be useless to discuss them ; they all strike the reviewer at once as being clear, complete and thoroughly modern.

The contributions to syphilis which appear are the views as represented by many of our most noted authorities, and deals with the subject as subjoined : Acquired syphilis, by James S. Howe. Syphilis of the bones, joints, bursæ, tendons and muscles, by Lewis C. Bosher. Syphilis of the respiratory, circulatory, lymphatic and alimentary systems, by John H. Linsley. Syphilis of the nervous system, by Graeme M. Hammond. Syphilis of the eye, by Thomas R. Pooley. Heieditary syphilis, by Abraham Jacobi. Treatment of syphilis, by Robert Holmes Green. Chancroids, by James S. Howe.

In the first portion on Acquired syphilis the subject is honestly met, and the difficulties of differential diagnosis clearly shown. The portion devoted to bones, joints and the like is given in detail in connection with the latest and most scientific methods of treatment. The part of the subject-matter contributed by Dr. Linsley has special merit, being precise without being over-dogmatic ; full, but not burdensome. The increasing importance of syphilitic affections of the nervous system and of the eye meets with a corresponding recognition in this work.

Dr. Jacobi's article on hereditary syphilis exhibits facts and opinions that are logical and judicious with all its consequences to mother and child. The portion on treatment is concise, contains nothing new, but the collection of formulæ have been painstaking in their selection and cannot fail to be of value to anyone who has experience enough to enable him to select what he wants.

That section of the volume devoted to the consideration of Diseases of the skin is divided into two parts. Part I., Introduction, which includes the anatomy and physiology of the skin, general symptomatology, general etiology, general pathology, general diagnosis and classification. Part II., Special, is arranged into classes:

Class I., Inflammations; Class II., Hemorrhages; Class III., Hypertrophies; Class IV., Atrophies; Class V., New growths; Class VI., Neuroses; Class VII., Diseases of the appendages of the skin; Class VIII., Parasitic diseases.

It would be useless to here state the subdivision of each class, as it would entail too much space. However, the diseases most frequently met with are treated at the greatest length. The clinical descriptions are excellent; sound principles of treatment are laid down and numerous useful prescriptions are given. Among the numerous contributors the names of W. R. Robinson, W. A. Hardaway, J. A. Fordyce, A. Van Harlingen, G. T. Jackson and others of equal fame may be found.

On the whole, the volume contains 1,229 well-printed pages. Its contents are wonderfully complete. The colored illustrations are, most of them, well executed, some of them exceptionally so, and the woodcuts are good. E. W.

